# Report of Mortality in Buffalo for the Month of Sept., 1856

**Published:** 1856-11

**Authors:** C. L. Dayton

**Affiliations:** Health Physician


ART. VI.—*Report of Mortality in Buffalo for the month of Sept.,* 1856. By C. L. DAYTON, M. D., Health Physician.

| DISEASES. | No. | Males. | Females. |
|---|---|---|---|
| Accidens, | 8 | 7 | 1 |
| Asphyxia Immersorum, | 6 | 5 | 1 |
| Albuminaria et morbus cordis, | 1 | 1 | |
| Apoplexia, | 1 | 1 | |
| Cholera Infantum, | 9 | 5 | 4 |
| Cynanche Trachealis, | 1 | 1 | |
| Cancer of the stomach, | 1 | 1 | |
| Dysentery, | 18 | 10 | 8 |
| Diarrhœa, | 7 | 4 | 3 |
| Dentitio, | 8 | 4 | 4 |
| Debilitas Senilis, | 4 | 4 | |
| Eclampsia, | 5 | 3 | 2 |
| Effusion at the base of the Brain, | 1 | 1 | |
| Febris Typhus, | 1 | 1 | |
| " Scarlatina, | 5 | 5 | |
| " Scarlatina Maligna, | 1 | | 1 |
| " Pulmonalis, | 1 | 1 | |
| " Typhoid, | 4 | 3 | 1 |
| " Intermittent, | 1 | | 1 |
| " Gastrica, | 1 | 1 | 1 |
| Gastritis, | 1 | | 1 |
| Hyperæmia Cerebralis, | 2 | | 1 |
| Hydrocephalus Acutus, | 3 | 1 | 2 |
| " Chronica, | 2 | 1 | |
| Hydrops, | 3 | 3 | |
| Hepatitis Chronica, | 1 | 1 | |
| In emperance, | 4 | 2 | 2 |
| Ignotus, | 16 | 12 | 4 |
| Mania a Potu, | 1 | 1 | |
| Marasmus, | 10 | 7 | 3 |
| Murder, | 1 | | 1 |
| Morbus Hepatis, | 1 | | 1 |
| " Cordis, | 1 | 1 | |
| Premature Birth, | 1 | | 1 |
| Paralysis, | 1 | 1 | |
| " Produced by softening of the Brain, | 1 | | 1 |
| Pneumonitis, | 2 | 1 | 1 |
| " Typhoides, | 2 | 2 | |
| Phlebitis et Diarrhœa, | 1 | | 1 |
| Poisoned by Arsenic, | 1 | 1 | |
| Scrophula, | 1 | | 1 |
| Still-born, | 10 | 5 | 3 |
| Summer Complaint, | 26 | 17 | 9 |
| Tuberculosis Pulmonalis, | 18 | 4 | 14 |
| " Cerebralis, | 3 | 3 | |
| " et Diarrhœa Chronica, | 1 | 1 | |
| Ulceration of the Bowels, | 1 | 1 | |
| Total, | 199 | | |

### SEXES.

| | |
|---|---|
| Males, | 123 |
| Females, | 73 |
| Sex not given, | 3 |
| Total, | 199 |

### AGES.

| | | | |
|---|---|---|---|
| Still-born, | 10 | Between 10 years and 20 years, | 6 |
| 1 day, | 0 | " 20 " " 30 " | 13 |
| Between 1 day and 30 days, | 10 | " 30 " " 40 " | 23 |
| " 1 month and 6 months, | 24 | " 40 " " 50 " | 8 |
| " 6 months and 12 " | 34 | " 50 " " 60 " | 8 |
| " 1 year " 3 years, | 41 | " 60 " " 70 " | 5 |
| " 3 " " 5 " | 0 | " 70 " " 80 " | 5 |
| " 5 " " 10 " | 6 | " 80 " " 90 " | 1 |
| Total number under 10 years, | 125 | Total number over 10 years, | 69 |
| Ages not given, | 5 | Total, | 199 |

### NATIVITIES.

| | | | |
|---|---|---|---|
| American, (white, 76), colored, 3, | 79 | Prussian, | 3 |
| German, | 55 | French, | 2 |
| Irish, | 23 | Scotch, | 0 |
| English, | 7 | Italian, | 0 |
| Canadian, | 3 | Country not given, | 27 |
| | | Total, | 199 |

---

*Gestation prolonged beyond the natural term by Homœopathic means.*—The Gazette Hebdomadaire, of Paris, mentions, in a sarcastic strain, a little bit of Hahnemannian sorcery recently perpetrated in the French capital. Puerperal fever having been rife of late, many ladies on the eve of parturition were in great alarm; one, however, expressed herself with great confidence on the subject, saying that her homœopathic attendant was giving her certain globules, by means of which her confinement would not take place at the accustomed period, but *would be delayed* until the epidemic had abated.—*London Lancet.*

---

*Strychnia.*—Dr. Fleetwood Churchill, calls attention, in the English journals, to an error in his work on *Diseases of Females*, which might lead to serious consequences. He states, in the work alluded to, in treating of amenorrhœa, that strychnia has been given advantageously for its cure, in the dose of from one-tenth of a grain to a grain, three or four times a day. The latter dose would be unquestionably a poisonous one. The dose should be from one sixteenth to one twelfth of a grain, and it would not be wise to give more.

